# Subsidized gestational diabetes mellitus screening and management program in rural China: a pragmatic multicenter, randomized controlled trial

**DOI:** 10.1186/s12916-024-03330-1

**Published:** 2024-03-05

**Authors:** Tingting Xu, Qing Xia, Xiaozhen Lai, Kun He, Dazhi Fan, Liangkun Ma, Hai Fang

**Affiliations:** 1 Department of Health Management and Policy, School of Public Health, Capital Medical University, Beijing, 100069 China; 2 School of Public Health, Peking University, Beijing, 100083 China; 3 Australian Centre for Health Services Innovation and Centre for Healthcare Transformation, School of Public Health & Social Work, Faculty of Health, Queensland University of Technology, Brisbane, QLD Australia; 4 Health Economics Research Centre, Nuffield Department of Population Health, University of Oxford, Oxford, UK; 5 National Children’s Medical Center, Children’s Hospital of Fudan University, Shanghai, 201102 China; 6 Foshan Fetal Medicine Research Institute, Affiliated Women and Children Hospital, Southern Medical University, Guangdong, 528000 China; 7 Department of Obstetrics, Affiliated Foshan Women and Children Hospital, Southern Medical University, Guangdong, 528000 China; 8 Department of Obstetrics and Gynecology, Peking Union Medical College Hospital, Beijing, China; 9 Institute for Global Health and Development, Peking University, Beijing, 100871 China

**Keywords:** Gestational diabetes mellitus, Subsidy, Randomized controlled trial, Maternal complications, Neonatal complications

## Abstract

**Background:**

The increasing prevalence of gestational diabetes mellitus (GDM) is a major challenge, particularly in rural areas of China where control rates are suboptimal. This study aimed to evaluate the effectiveness of a GDM subsidy program in promoting GDM screening and management in these underserved regions.

**Methods:**

This multicenter, randomized controlled trial (RCT) was conducted in obstetric clinics of six rural hospitals located in three provinces in China. Eligible participants were pregnant women in 24–28 weeks’ gestation, without overt diabetes, with a singleton pregnancy, access to a telephone, and provided informed consent. Participants were randomly assigned in a 1:1 ratio to either the intervention or control groups using an internet-based, computer-generated randomization system. The intervention group received subsidized care for GDM, which included screening, blood glucose retesting, and lifestyle management, with financial assistance provided to health care providers. In contrast, the control group received usual care. The primary outcomes of this study were the combined maternal and neonatal complications associated with GDM, as defined by the occurrence of at least one pre-defined complication in either the mother or newborn. The secondary outcomes included the GDM screening rate, rates of glucose retesting for pregnant women diagnosed with GDM, dietary patterns, physical activity levels, gestational weight gain, and antenatal visit frequency for exploratory purposes. Primary and secondary outcomes were obtained for all participants with and without GDM. Binary outcomes were analyzed by the generalized linear model with a link of logistic, and odds ratios (OR) with 95% confidence intervals (CIs) were reported. Count outcomes were analyzed by Poisson regression, and incidence rate ratios with 95% CIs were reported.

**Results:**

A total of 3294 pregnant women were randomly assigned to either the intervention group (*n* = 1649) or the control group (*n* = 1645) between 15 September 2018 and 30 September 2019. The proportion of pregnant women in the intervention group who suffered from combined maternal and/or neonatal complications was lower than in the control group with adjusted OR = 0.86 (0.80 to 0.94, *P* = 0.001), and a more significant difference was observed in the GDM subgroup (adjusted OR = 0.66, 95% CI 0.47 to 0.95, *P* = 0.025). No predefined safety or adverse events of ketosis or ketoacidosis associated with GDM management were detected in this study. Both the intervention and control groups had high GDM screening rates (intervention: 97.2% [1602/1649]; control: 94.5% [1555/1645], *P* < 0.001). Moreover, The intervention group showed a healthier lifestyle, with lower energy intake and more walking minutes (*P* values < 0.05), and more frequent blood glucose testing (1.5 vs. 0.4 visits; *P* = 0.001) compared to the control group.

**Conclusion:**

In rural China, a GDM care program that provided incentives for both pregnant women and healthcare providers resulted in improved maternal and neonatal health outcomes. Public health subsidy programs in China should consider incorporating GDM screening and management to further enhance reproductive health.

**Trial registration:**

China Clinical Trials Registry ChiCTR1800017488. https://www.chictr.org.cn/

**Supplementary Information:**

The online version contains supplementary material available at 10.1186/s12916-024-03330-1.

## Background

Gestational diabetes mellitus (GDM) is a common pregnancy-related condition, defined as the onset of diabetes during the second or third trimester rather than pre-existing diabetes before pregnancy [1]. The prevalence of GDM has significantly increased globally and in China over the last two decades [2, 3]. In 2020, it was estimated that 20.9 million pregnant women and their newborn babies worldwide were affected by GDM [2, 3], with over 2 million GDM pregnant women diagnosed in China, half of whom resided in rural areas [4]. GDM is significantly associated with an increased risk of various adverse outcomes, including postpartum hemorrhage, pre-eclampsia, neonatal hypoglycemia, macrosomia, cesarean section (CS), and maternal and neonatal mortality [5]. Moreover, GDM raises the likelihood of pregnant women and their offspring developing type II diabetes, obesity, and metabolic syndrome later in life [5, 6].

Lifestyle management, which includes dietary and physical activity interventions, is considered the foundation of GDM treatment. Clinical trials have shown that more than 80% of pregnant women with GDM can be effectively managed through lifestyle interventions during the third trimester [7, 8]. In 2020, a national guideline for GDM screening and management in China was issued, emphasizing the need for standardized GDM care for all pregnant women in China. This includes health education, GDM screening, lifestyle management (such as diet and exercise), reminders, blood glucose monitoring, and insulin therapy [9]. Nevertheless, the implementation of standardized GDM care remains suboptimal, particularly in rural areas of China [6, 9–12]. Numerous studies have reported inadequate GDM care in these areas, with screening rates below 50% and lifestyle management rates below 20% [3, 10–12]. Given China’s recent family planning policy that allows a maximum of three children per family, pregnant women of advanced maternal age and/or multiparity face an increased risk of developing GDM [13]. Therefore, promoting GDM care in rural China is crucial to prevent adverse maternal and neonatal outcomes, especially for this high-risk population.

Previous in-depth interviews and willingness-to-pay studies have identified two economic factors as major barriers to adequate GDM screening and management in rural China [12, 14, 15]. Firstly, GDM screening and management are not covered by maternal health insurance or public health programs in China, resulting in 100% out-of-pocket expenses. The majority of pregnant women in low-income areas with low socioeconomic status reported being unwilling to pay for these services [12, 14], which is exacerbated by their tendency to underestimate the risk of developing GDM and its adverse consequences based on their pre-conceptional health status [6, 14–16]. Secondly, standardized GDM care, including health education and telephone reminders, usually exceeds the routine responsibilities of health professionals in low-income hospital settings, leading to a lack of motivation to provide GDM care without financial incentives [17].

To date, no studies have examined the impact of complex interventions, including both financial incentives and standardized GDM care, on pregnant women, particularly in low- and middle-income countries [6, 18]. Previous investigations have primarily assessed the effects of standardized care on pregnancy outcomes in women with GDM, focusing on health education, dietary management, physical activity, medication therapy, or a combination of these interventions [19–21]. For example, systematic reviews and meta-analyses of randomized controlled trials (RCTs) have demonstrated that health education interventions significantly reduce GDM incidence and improve maternal and fetal outcomes [22, 23]. A range of dietary interventions, such as a low-carbohydrate diet, a low-glycemic-index diet, and a balanced diet, have been investigated and proven effective in enhancing glycemic control, reducing insulin therapy requirements, and improving maternal and fetal outcomes [24, 25]. Moreover, several RCTs conducted in China have demonstrated that supervised exercise programs effectively improve glycemic control and reduce insulin therapy requirements in pregnant women with GDM [26, 27]. Nevertheless, the screening and management rates for GDM in rural China continue to be low.

To address this research gap, this study aims to implement a financially subsidized GDM care program in rural areas of China and evaluate its effectiveness in promoting GDM screening and management, as well as improving maternal and neonatal health outcomes. We anticipate that this all-encompassing intervention will provide a pragmatic solution for managing GDM in rural China, by tackling the financial barriers to screening and management and providing standardized care to pregnant women.

## Methods

### Study design

The study focused on the impact of the subsidized intervention of GDM screening and management on the subsequent maternal and neonatal complications. We conducted an RCT in obstetric clinics located in six rural hospitals, with two counties located in each of the Shaanxi, Sichuan, and Yunnan provinces in China. This was a multicenter, prospective, and open-label trial that enrolled participants from 6 counties with Gross Domestic Product (GDP) per capita of less than 13,000 Chinese yuan (approximately 2000 US dollars in 2018) per year (as shown in Additional file 1: Fig. S1). We selected these study sites based on several factors: their representation of the western region in China, their recognition as impoverished counties, the presence of poor routine screening and management practices for GDM, and their similarity in approaches to GDM management. All six study hospitals were located in poverty counties defined by the Poverty Alleviation Office of the State Council in China. In 2018, the GDP per capita in Pingchang and Yingshan counties of Sichuan province was 12,800 yuan and 13,500 yuan, respectively; Hanyin and Ziyang counties in Shaanxi province recorded 14,500 yuan and 10,800 yuan; while Yiliang and Zhaotong counties in Yunnan province were 10,600 yuan and 21,000 yuan, respectively. These figures were significantly lower than the average GDP per capita of their respective provinces in 2018–48,883 yuan in Sichuan, 63,477 yuan in Shaanxi, and 37,136 yuan in Yunnan — demonstrating a similar status of poor GDM routine screening and management. Before the trial, the average screening and management rates across the six study sites were only 42% and 16%, respectively.

The trial was approved by the Ethics Committees of Peking University (IRB00001052-18052; Beijing, China), registered in the China Clinical Trials Registry (registration number: ChiCTR1800017488), and implemented in 15 September 2018–01 November 2021. The trial protocol and statistical analysis plan were published previously [28] and are available online. A study design flowchart is presented in Additional file 1: Fig. S2. Data collection followed the CONSORT guidelines for reporting randomized trials (see Additional file 1: CONSORT guidelines) [29].

### Participants and inclusion criteria

Healthcare providers involved in the study evaluated pregnant women who were at 24–28 weeks’ gestation to determine their eligibility based on the inclusion criteria. These assessments were carried out on a daily basis to identify eligible participants who visited the hospitals for routine antenatal care. The eligibility criteria for participants in this study were as follows: (1) pregnant women at 24–28 weeks’ gestation; (2) without overt diabetes (i.e., type I or type II diabetes) prior to pregnancy; (3) with a singleton pregnancy; (4) with access to a telephone; and (5) who provided written informed consent. The exclusion criteria for this study were as follows: (1) Participants with fasting blood glucose levels above 7 mmol/L or glycosylated hemoglobin levels equal to or greater than 6.5% during the first GDM screening were excluded to eliminate pregnant women with manifest diabetes prior to pregnancy. (2) Individuals with concomitant serious systemic diseases, such as essential hypertension, renal disease, thalassemia, systemic lupus erythematosus, celiac disease, or thyroid disease, were also excluded to prevent the confounding influence of severe systemic diseases on the intervention outcomes of this study; and (3) individuals with physical or cognitive disabilities.

During the informed consent process, pregnant women were provided with clear information regarding their potential participation in the study. They were informed that they would be randomized to either the intervention group or the control group with different costs, subsidies, and services provided by hospitals. All participants who took part in the outcome assessments gave written informed consent.

### Randomization

To ensure a robust methodology, eligible pregnant participants were enrolled in the study and randomly assigned to either the intervention or control group at a 1:1 ratio using an internet-based computer-generated randomization system with concealed allocation. The randomization sequence was stratified by the six study hospital sites, and balanced blocks of six participants were utilized. A skilled outpatient nurse performed the allocation of participants. Due to the nature of the trial interventions, neither the medical staff nor the pregnant participants were blinded to the allocation.

### The intervention group

Participants in the intervention group received subsidized GDM care, which included GDM health education, GDM screening using a 75-g oral glucose tolerance test (OGTT) [30], lifestyle management (including diet and exercise), telephone reminders, and follow-up blood glucose retesting (fasting glucose test and 2-h-postprandial glucose test). The financial subsidy ensured that pregnant women received the aforementioned GDM care free of charge, and the costs were later reimbursed by the project funding. The healthcare providers were provided with financial incentives of 10 Chinese yuan per participant for offering health education and guidance on GDM screening, and an additional 20 Chinese yuan per GDM patient for providing lifestyle management, telephone reminders, and guidance on follow-up blood glucose testing in the intervention group.

### The control group

Participants in the control group received usual care, which was consistent across all six trial sites. This typically involved a basic reminder about GDM screening for pregnant women, recommendations on diet and exercise for women diagnosed with GDM, while participants took their routine antenatal care. No financial incentives or subsidies were provided to either pregnant women or healthcare providers in the control group.

### Procedures

The detailed trial process is presented in Additional file 1: Fig. S2 and described in our published protocol [28]. Two experienced nurses were in charge of explaining the trial’s purpose and enrolling pregnant women in their 24–28 weeks of gestation. A self-administered questionnaire was used at the time of recruitment, before randomization, to collect information on participants’ sociodemographic characteristics, knowledge of GDM, and their diet and physical activity in the two weeks preceding the questionnaire. To ensure accurate and reliable data collection, trained study staff members were available to provide support when needed. If a pregnant woman encountered difficulties understanding the questionnaire or faced challenges in completing it on her own, a nurse would offer guidance and clarification to facilitate the process. Additionally, for participants who were illiterate, the nurse would assist them in completing the forms.

The aim of providing subsidized GDM care in the intervention group was to encourage participants and healthcare professionals to adhere to the standardized GDM care recommended by GDM guidelines in China. Each intervention participant received a free health education session of at least 10 min, which covered topics such as the concept of GDM, risk factors, complications, screening procedures, and dietary and physical activity recommendations. Additionally, a handbook with the same content was provided to participants. During the session, the intervention nurse informed participants that they could undergo a free GDM screening using a 75-g OGTT at 24–28 weeks of gestation.

Participants diagnosed with GDM were granted immediate and complimentary access to a 30-min individual education session on diet and exercise self-monitoring. These sessions, tailored for women in the GDM intervention group, aimed to bolster adherence to the dietary and exercise recommendations provided by clinical obstetricians. In tandem, detailed dietary guidelines were issued (Additional file 1: Supporting Materials), featuring illustrations that vividly depict food choices and portion sizes. Additionally, exercise guidelines, presented in a graphical format, were incorporated into the intervention (Additional file 1: Supporting Material). To assist healthcare providers in customizing diet and exercise advice for each GDM patient, a logbook was distributed (Additional file 1: Supporting Material). This logbook, designed for daily recording of diet and exercise, was to be filled out by the participants and reviewed by their obstetricians during prenatal visits, thereby enabling real-time adjustments based on the individual conditions of the pregnant women. Moreover, follow-up blood glucose retesting (including fasting glucose test and 2-h postprandial glucose test) was offered free of charge initially in the first two weeks, followed by subsequent tests every four weeks up until 34 weeks of gestation.

If a participant missed her glucose retest during follow-up, nurses provided a reminder via telephone or through the social media communication application WeChat. For pregnant women without GDM, weekly remote diet and exercise advice and management were provided through WeChat to reduce the potential risk of abnormal glucose in the last trimester. Moreover, healthcare providers in the intervention group were financially incentivized with 10 Chinese yuan (equivalent to 1.51 US dollars in 2018) per participant for providing health education and guidance on GDM screening, and with 20 Chinese yuan per GDM patient to provide lifestyle management, telephone reminders, and guidance on follow-up blood glucose testing.

The control group participants received usual care without any subsidies or financial incentives, and the behavior of healthcare professionals in the control group was not influenced. However, GDM screening, lifestyle management, and follow-up blood glucose testing were available to the control group participants if they paid for it out of pocket. Nevertheless, they did not have access to formal GDM health education or telephone reminders, which were exclusively provided to the intervention group.

Both groups were reassessed after 34 weeks of gestation to evaluate their knowledge of GDM, two-week diet, and physical activity for any changes. A medical record was maintained for each participant in both groups from enrollment until delivery, including information on GDM screening, prevalence of GDM, follow-up blood glucose retesting, mode of delivery, birth weight, and maternal and infant complications. Participants transferred to a higher level of hospital for various reasons were still followed up for their maternal and neonatal outcomes. In this individual-based RCT, pregnant women from both groups were intentionally not grouped together in the same location to reduce the chances of information exchange. We provided rigorous training for healthcare providers involved in the study to ensure standardized procedures and data collection. Regular monitoring and supervision were conducted by the research team to ensure adherence to the study protocol in both intervention and control groups.

### Outcomes

We pre-specified primary outcomes that focused on combined maternal and neonatal complications related to GDM. To identify these outcomes, we conducted a comprehensive review of the existing literature and identified 15 distinct types of maternal complications and 14 distinct types of neonatal complications associated with GDM (refer to Additional file 1: Table S1).

Furthermore, we defined a set of secondary outcomes for exploratory purposes, including the GDM screening rate, glucose retesting for pregnant women diagnosed with GDM, dietary patterns, physical activity levels, gestational weight gain, antenatal visit frequency, and laboratory measurements. These secondary outcomes were established to provide a comprehensive evaluation of the impact of GDM care on maternal and neonatal health, as well as to explore potential avenues for intervention and prevention.

### Safety and adverse events

The safety monitoring and adverse event assessment in this study, pertaining to GDM management, focused on the occurrence of ketosis and/or ketoacidosis, because all maternal and neonatal complications had been utilized as the primary outcomes. Urine and/or ketone testing were conducted for enrolled participants who presented clinical symptoms during the baseline enrollment and/or monthly antenatal checkups. Both urine and blood ketone testing were performed for all participants during delivery. Pregnant women who meet the diagnostic criteria for clinical ketosis and ketoacidosis, regardless of their assigned group, will be referred to a higher-level hospital for additional testing. Those diagnosed with ketosis or ketoacidosis by the physician will receive treatment and be withdrawn from the study. Pregnant women who were not diagnosed with clinical ketosis or ketoacidosis but were referred to higher-level hospitals other than the original study site were contacted via phone calls for survey purposes. Follow-up data was collected on blood glucose levels, childbirth, and the maternal and child health status.

### Statistical analysis

To determine the sample size for the RCT, three outcome measures were considered: the GDM screening rate, the rate of cesarean section, and the incidence of macrosomia. It was hypothesized that the subsidized intervention would significantly increase the GDM screening rate. Based on previous studies, the screening rate in rural China was assumed to be 50%. The primary outcome of this study was the overall incidence of maternal and neonatal complications. Since the overall incidence of maternal and neonatal complications in China was not available, sample size calculation was based on the rates of cesarean section and macrosomia, which were the major complications related to GDM in maternal women and neonatal babies, respectively. To ensure 80% statistical power to detect a 25% increase for screening rate (from 50 to 62.5%), a 40% relative risk reduction for cesarean section (from 51.4 to 30.5%) [10, 19], and an 80% relative risk reduction for macrosomia (from 6.2% to 1.2%) [19, 31], we calculated that 440 pregnant women would need to be enrolled. Considering the prevalent rate of GDM in rural areas of China as 17.5% and 20% dropout, the sample size was calculated to be no less than 3136 pregnant women.

All analyses were conducted using the intention-to-treat principle and were performed with Stata software (STATA 17.0, Stata Corp, College Station, TX, USA). Descriptive statistics were run for all variables, with continuous and binary variables presented as mean (standard deviation, SD) and proportion (numbers), respectively. Intervention effects for binary outcomes were analyzed with odds ratios (OR) and 95% confidence intervals (CIs), and count measurements were expressed as mean differences with 95% CIs. Independent samples *t*-tests and *χ*2 or Fisher’s exact tests were used as appropriate. Statistical significance was set at *P* ≤ 0.05 with two-sided. For the analysis of primary and secondary outcomes, we further conducted a generalized linear model with a link of logistic and Poisson regression model to analyze the intervention effect, adjusting for 6 clusters of study hospitals. We also used a Difference-in-Difference (DID) model to assess the intervention effect on improving dietary patterns and physical activity levels for exploratory purposes. All analysis of primary and secondary outcomes was also stratified by GDM. A Bonferroni correction was employed for the multiple testing of the entire study sample and the subgroup of participants with GDM. No imputation technique for primary outcomes was used as the participants with missing maternal or neonatal outcomes were balanced in their characteristics, and only 3.4% (*n* = 56) in the treatment group and 3.0% (*n* = 50) in the control group. To assess safety, adverse events (AEs) will be analyzed by determining the number of occurrences and the proportion of participants who experienced AEs in both the intervention and control groups. A Data Safety Monitoring Board was involved in overseeing the implementation of this trial.

## Results

### Participants

**Fig. 1:**
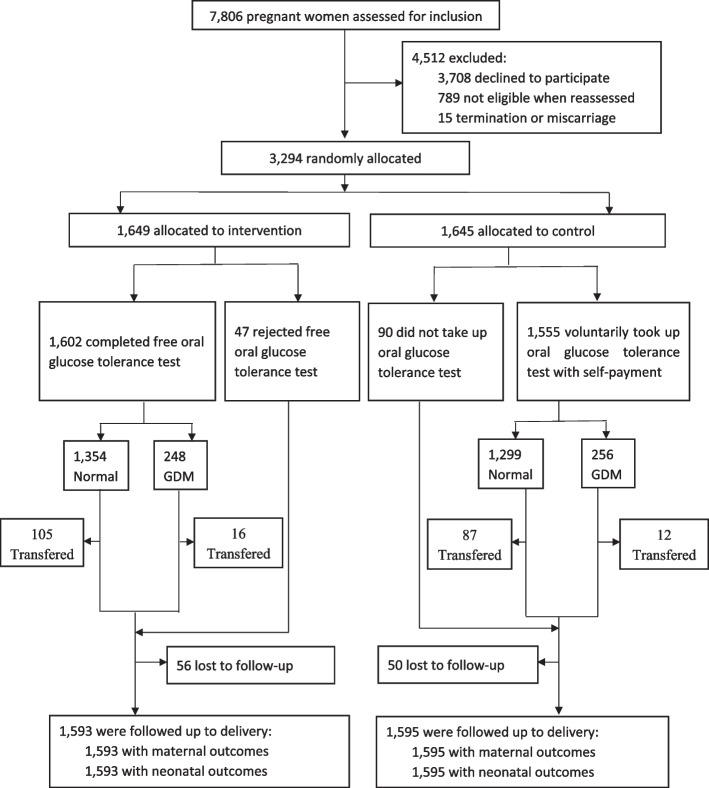
Randomized controlled trial profile

During this trial, a total of 7806 pregnant women in their 24–28th week of gestation between 15 September 2018 and 30 September 2019 were assessed. Of these women, 3,294 (42%) were randomly allocated to either the intervention group (*n* = 1649) or the control group (*n* = 1645). Ultimately, 1593 (96.6%) pregnant women in the intervention group and 1595 (96.9%) in the control group were followed up until delivery, as shown in Fig. 1. The baseline characteristics of the intervention and control groups were comparable, as detailed in Table 1. The mean (SD) age of the enrolled women was 27.1 (5.0) years, and their mean pre-pregnancy BMI was 21.1 (3.5) kg/m^2^. Among the participants, 34.2% were primiparous, 60.1% had a lower educational level (middle school or below), and the mean annual household income was 6.1 (5.9) ten thousand Chinese yuan. As indicated in Additional file 1: Table S2, the baseline characteristics of pregnant women who were included in the analysis were also comparable with those who were lost to follow-up.

**Table 1:** Baseline characteristics at recruitment

	**Overall (3294)**	**Intervention**			**Control**		
		**Overall (** ***N*** ** = 1649)**	**Normal (** ***n*** ** = 1354)**	**GDM (** ***n*** ** = 248)**	**Overall (** ***N*** ** = 1645)**	**Normal (** ***n*** ** = 1299)**	**GDM (** ***n*** ** = 256)**
Maternal age, *years, mean (SD)*	27.1(5.0)	27.0 (5.0)	26.6 (4.8)	28.6 (5.4)	27.2 (5.0)	26.9 (4.8)	29.1 (5.5)
Pre-pregnancy BMI, *kg/m* ^*2*^ *, mean (SD)*	21.1(3.5)	21.1 (3.7)	20.8 (3.3)	22.5 (4.3)	21.2 (3.3)	20.9 (3.1)	22.1 (3.8)
Gestational weeks at recruitment, weeks,* mean (SD)*	25.4(1.5)	25.4 (1.5)	25.3 (1.4)	25.6 (1.5)	25.4 (1.5)	25.3 (1.4)	25.7 (1.7)
Primiparous, No. (%)	991(34.2)	500 (33.8)	432 (35.0)	62 (28.3)	491 (34.7)	407 (35.6)	63 (29.03)
Fasting blood glucose, mmol/L, mean (SD)	4.4(0.6)	4.4 (0.6)	4.3 (0.5)	5.2 (0.7)	4.5 (0.6)	4.3 (0.4)	5.3 (0.6)
One-hour blood glucose, mmol/L, mean (SD)	6.9(1.8)	6.8 (1.8)	6.4 (1.4)	8.9 (2.2)	7.0 (1.8)	6.6 (1.4)	9.2 (2.2)
Two-hour blood glucose, mmol/L, mean (SD)	6.1(1.4)	6.0 (1.4)	5.8 (1.0)	7.6 (2.0)	6.2 (1.4)	5.9 (1.1)	7.7 (1.7)
Systolic blood pressure before pregnancy, *mmHg, mean (SD)*	110.0 (11.4)	110.0 (11.5)	109.4 (11.4)	113 (12.0)	110.2 (11.4)	109.7 (11.1)	112.0 (12.9)
Diastolic blood pressure before pregnancy, *mmHg, mean (SD)*	68.3(10.5)	68.5 (10.9)	68.2 (10.8)	69.6 (8.6)	68.1 (10.2)	67.8 (10.3)	69.5 (10.0)
Education, *No. (%)*							
*Below primary school*	*41 (1.4)*	23 (1.5)	15 (1.2)	7 (3.1)	18 (1.2)	12 (1.0)	4 (1.8)
*Primary school*	*335 (11.3)*	180 (11.9)	150 (11.9)	29 (12.7)	155 (10.7)	120 (10.2)	31 (14.0)
*Junior high school*	*1408 (47.4)*	738 (48.6)	623 (49.3)	101 (44.3)	670 (46.1)	555 (47.3)	93 (41.9)
*Senior high school*	*332 (11.3)*	166 (10.9)	142 (11.3)	22 (9.6)	166 (11.4)	130 (11.1)	27 (12.1)
*Technical secondary school*	*260 (8.7)*	132 (8.7)	110 (8.7)	17 (7.5)	128 (8.8)	108 (9.2)	16 (7.2)
*Two year’s college*	*345 (11.6)*	168 (11.0)	140 (11.1)	26 (11.4)	177 (12.2)	141 (12.0)	28 (12.6)
*Four years of university or above*	*251 (8.5)*	112 (7.4)	85 (6.7)	26 (11.4)	139 (9.6)	108 (9.2)	23 (10.4)
Family income per year, 10 thousand Chinese yuan							
*Mean(SD)*	*6.1 (5.9)*	*5.9 (4.8)*	6.0 (5.0)	5.7 (3.5)	6.2 (6.8)	6.1(7.2)	6.5 (5.3)
*Median (the interquartile range)*	*5.0 (3;8)*	*5.0 (3;8)*	*5.0 (3;8)*	*5.0 (3;8)*	*5.0 (3;8)*	*5.0 (3;8)*	*5.0 (3;8)*
*Fanmily size*	*4.2 (1.5)*	*4.2 (1.4)*	4.2 (1.4)	3.9 (1.4)	4.2 (1.6)	4.2(1.6)	3.9 (1.5)
*The incidence rate of GDM, n/No. (%)*^a^	*504/3157 (16.0)*	248/1602 (15.5)			256/1555 (16.5)		
*The uptake rate of GDM screening, No. (%)*		1602 (97.2)			1555 (94.5)		

Footnote**:** The baseline characteristics are not statistically different between intervention and control groups by *t*-test or chi-square test

*Abbreviations*: *BMI* Body mass index, *S.D.* Standard deviation, *GDM* Gestational diabetes mellitus

^a^1602 (97.2% out of 1649) participants in the intervention group and 1555 (94.5% out of 1645) participants in the intervention group took up GDM screening

### Primary outcomes: maternal and neonatal complications

Table 2 displays the incidence of maternal and neonatal complications related to GDM in the intervention and control groups. Overall, the incidence of combined maternal or neonatal complications was slightly lower in the intervention group compared to the control group (38.6% vs. 42.0%, OR 0.86 [0.80 to 0.94] in the generalized linear model with a link of logistic). However, in the subgroup of participants with GDM, the difference between the two groups was more significant (38.8% vs. 48.6%, OR 0.66 [0.47 to 0.95]). Similar results were obtained in the number of combined maternal and neonatal complications, 0.50 vs. 0.54, and the Poisson regression model produced an incidence rate ratio (IRR) of 0.93 (95% CI, 0.87 to 0.99) in the entire sample and 0.86 (95% CI, 0.67 to 1.10) in the subgroup of participants with GDM.

**Table 2:** Maternal and neonatal complications between the intervention group and the control group

	**Intervention Group**			**Control Group**			**Intervention effect for all pregnant women** ^**a**^		**Intervention effect for pregnant women with GDM** ^**a**^	
	Overall (*N* = 1649)	Normal (*n* = 1354)	GDM (*n* = 248)	Overall (*N* = 1645)	Normal (*n* = 1299)	GDM (*n* = 256)				
							OR (95% CI)	*P* value	OR (95% CI)	*P* value
Maternal or neonatal complications, *N* (%)	615 (38.6)	507 (38.6)	93 (38.8)	669 (42.0)	524 (41.0)	121 (48.6)	0.86 (0.80 to 0.94)	0.001	0.66 (0.47 to 0.95)	0.025
							IRR (95%CI)	*P* value	IRR (95% CI)	*P* value
The number of maternal and neonatal complications per person, mean (SD)	0.50(0.74)	0.50(0.73)	0.51 (0.76)	0.54 (0.74)	0.52 (0.73)	0.60 (0.70)	0.93 (0.87 to 0.99)	0.035	0.86 (0.67 to 1.10)	0.244

All 29 maternal and neonatal complications were included into the combined maternal and neonatal complications in Table 2 to provide a comprehensive overview of the primary outcomes

We utilized generalized linear models with a logistic distribution link to analyze maternal and neonatal complications. Additionally, a Poisson regression model was employed to assess the number of maternal and neonatal complications per person

*Abbreviations*: *GDM* Gestational diabetes mellitus, *OR* Odds ratio, *IRR* Incidence rate ratio

^a^The intervention effect for all pregnant women or pregnant women with GDM was adjusted for hospital, and a statistical significance level of *P* < 0.05 (two-sided) was used for all pregnant women, while a significance level of *P* < 0.025 (with Bonferroni correction) was used for pregnant women with GDM

For exploratory purposes, we found that the intervention group had lower probabilities (OR = 0.82, 95% CI 0.76 to 0.88) and smaller numbers (IRR = 0.83, 95% CI 0.79 to 0.87) of neonatal complications than the control group in the entire sample (Additional file 1: Table S3). The most commonly observed specific maternal complications among GDM women in both groups were cesarean Sect. (30.0% vs. 37.0%) and premature delivery (4.2% vs. 5.2%) (Additional file 1: Table S4). The most common neonatal complications were fetal macrosomia (3.8% vs. 5.6%) and neonatal jaundice (2.9% vs. 4.0%). Additional file 1: Table S5 presented other less frequent maternal and neonatal complications. Additional file 1: Table S6 reported results from 3 study provinces. Moreover, neither the intervention nor the control group experienced instances of starvation ketosis, ketoacidosis, or adverse events that could be medically attributed to GDM management in this study.

### Secondary outcomes: GDM screening uptake rates

The uptake rate of GDM screening was high in both the intervention and control groups. In the intervention group, 97.2% (1602/1649) of pregnant women underwent free GDM screening after receiving health education at the trial’s onset. Meanwhile, 95.5% (1555/1645) of participants in the control group underwent GDM screening with a self-payment of 100% (*P* < 0.001), as presented in Table 1. The overall incidence rate of GDM in rural areas of China was observed to be 16.0%, with 15.5% (248/1602) in the intervention group and 16.5% (256/1555) in the control group (*P* = 0.45).

### Maternal dietary patterns and physical activities

Table 3 At baseline, a comparison of the daily nutrition and physical activities between the intervention and control groups revealed no significant difference. However, following the intervention, pregnant women in the intervention group had a lower energy intake (DID, − 217.8 (SD, 87.6) kcal/day, *P* < 0.013) than those in the control group by consuming less carbohydrate (including rice and wheat flour), while increasing their vegetable intake. Similar findings were observed in the consumption of aquatic products and eggs in both groups. No significant difference was found between the groups in terms of other food intake (i.e., poultry/livestock meat, beans products, and beverages) or the distribution of total energy intakes in GDM groups. Although no difference was found in vigorous and moderate activities between the intervention and control groups, women in the intervention group were found to spend an additional 5.1 (SD 2.3, *P* = 0.025) min on walking.

**Table 3:** Maternal dietary patterns and physical activity between the intervention group and the control group at baseline and follow-up

	**Intervention**				**Control**							
	**Baseline**		**Follow-up**		**Baseline**		**Follow-up**		**Overall women**		**GDM women**	
	**Overall (** ***N*** ** = 1649)**	**GDM (** ***N*** ** = 248)**	**Overall (** ***N*** ** = 1649)**	**GDM (** ***N*** ** = 248)**	**Overall (** ***N*** ** = 1645)**	**GDM (** ***N*** ** = 256)**	**Overall (** ***N*** ** = 1645)**	**GDM (** ***N*** ** = 256)**	**DID** ^a^	***P*** ** value**	**DID** ^**a**^	***P*** ** value**
**Dietary pattern**												
Total energy intake, kcal/day, mean (SD)	1781.6 (2166.6)	1924.6 (1195.8)	1757.9 (1555.8)	1712.2 (1775.4)	1750.2 (1343.9)	1963.1 (1705.0)	1971.9 (1486.6)	2024.4 (1652.3)	− 217.8 (87.5)	0.013	− 121.9 (230.9)	0.596
Carbohydrate, kcal/day, mean (SD)	1310.6 (1481.5)	1368.0 (868.2)	1260.1 (1035.4)	1189.4 (1269.6)	1250.7 (826.6)	1387.5 (1063.6)	1403.1 (1006.3)	1457.6 (1090.2)	− 171.8 (61.3)	0.005	27.9 (166.3)	0.867
Protein, kcal/day, mean (SD)	256.3 (379.8)	263.5 (158.7)	259.3 (222.6)	260.1 (239.4)	240.2 (193.9)	274.5 (248.6)	288.7 (202.1)	323.8 (222.6)	− 38.2 (14.1)	0.007	− 10 (31.7)	0.737
Total fat, kcal/day, mean (SD)	360.4 (494.8)	344.8 (312.2)	338.9 (407.3)	353.4 (376.1)	357.7 (459.6)	424.7 (595.7)	393.6 (385.9)	481.1 (461.7)	− 45.7 (23.8)	0.055	− 11.6 (63.9)	0.856
Carbohydrate, % energy	69.5 (13.3)	69.3 (13.6)	68.2 (10.7)	65.6 (12.7)	70.0 (12.8)	68.4 (13.5)	67.5 (10.6)	64.9 (11.5)	0.6 (0.6)	0.363	1.0 (1.8)	0.558
Protein, % energy	13.0 (4.2)	13.7 (5.0)	14.2 (3.4)	15.1 (3.9)	12.8 (4.0)	13.4 (4.2)	14.1 (3.2)	14.6 (6.2)	0.1 (0.2)	0.793	0.2 (0.6)	0.728
Total fat, % energy	17.5 (10.8)	17.0 (10.1)	17.4 (8.8)	19.2 (10.5)	17.2 (10.5)	18.1 (11.1)	18.3 (8.8)	20.4 (9.8)	− 0.7 (0.5)	0.20	− 1.3 (1.5)	0.372
**Food intake amount/day**												
Rice, g, mean (SD)	218.9 (293.7)	252.2 (176.1)	207.6 (172.3)	187.6 (185.0)	204.9 (150.2)	224.8 (196.3)	243.7 (200.0)	246.6 (209.7)	− 45.8 (12.0)	< 0.001	− 7.2 (35.1)	0.838
Wheat flour, g, mean (SD)	42.9 (67.0)	49.6 (67.8)	47.9 (67.3)	47.8 (82.1)	43.6 (61.3)	54.6 (84.7)	55.6 (71.2)	61.1 (86.3)	− 6.0 (3.5)	0.085	1.5 (10.7)	0.885
Coarse cereals, g, mean (SD)	30.6 (172.8)	25.0 (43.6)	41.8 (68.9)	48.6 (81.7)	26.4 (53.1)	26.4 (139.1)	41.3 (60.0)	46.4 (66.9)	− 3.7 (5.5)	0.502	3.7 (8.7)	0.670
Potato flour, g, mean (SD)	58.0 (97.8)	57.8 (95.8)	45.8 (86.6)	45.3 (127.2)	58.7 (98.4)	66.4 (139.1)	51.2 (69.4)	49.2 (70.1)	− 4.3 (4.9)	0.383	5.8 (15.9)	0.717
Fried foods, g, mean (SD)	9.3 (39.7)	9.6 (30.9)	10.7 (31.6)	10.1 (28.5)	8.4 (25.0)	7.8 (26.0)	12.1 (34.4)	16.1 (59.6)	− 2.2 (1.7)	0.23	− 7.7 (5.3)	0.143
Vegetables, g, mean (SD)	134.9 (182.4)	141.0 (164.0)	262.7 (232.8)	275.5 (266.3)	136.2 (189.3)	177.5 (248.5)	179.1 (213.8)	187.5 (227.6)	84.8 (10.8)	< 0.001	119.9 (31.9)	< 0.001
Fruits, g, mean (SD)	226.5 (3398.0	212.2 (215.7)	164.8 (236.7)	171.9 (319.1)	217.3 (318.0)	247.4 (589.0)	151.8 (215.7)	128.6 (225.3)	7.4 (15.8)	0.640	105.6 (54.1)	0.05
Poultry/livestock meat, g, mean (SD)	69.5 (191.7)	77.5 (99.1)	60.6 (94.3)	57.3 (72.6)	65.7 (106.4)	81.9 (172.2)	64.1 (73.1)	73.2 (72.0)	− 4.6 (6.9)	0.501	8.2 (15.4)	0.594
Aquatic products, g, mean (SD)	27.8 (197.1)	23.8 (57.5)	23.8 (46.4)	26.3 (49.1)	21.4 (58.5)	20.4 (41.9)	34.8 (58.1)	42.6 (84.4)	− 17.4 (5.9)	0.003	− 19.5 (8.4)	0.021
Eggs, g, mean (SD)	45.5 (169.0)	43.2 (53.2)	42.8 (52.0)	50.3 (74.0)	39.0 (79.0)	41.5 (55.9)	52.1 (58.3)	60.7 (73.6)	− 15.6 (5.6)	0.005	− 7.2 (9.3)	0.446
Dairy products, g, mean (SD)	115.9 (182.0)	118.6(151.5)	139.3 (163.6)	138.0 (173.1)	125.1 (185.7)	133.2 (137.9)	132.0 (140.8)	139.6 (160.6)	15.9 (9.1)	0.080	16.6 (21.9)	0.446
Beans products, g, mean (SD)	44.2 (86.8)	53.4 (90.4)	42.5 (73.0)	51.0 (87.3)	41.5 (71.1)	54.5 (109.0)	47.9 (68.3)	49.2 (65.3)	− 6.8 (4.1)	0.090	11.7 (12.5)	0.350
Nuts, g, mean (SD)	31.9 (57.0)	29.8 (48.0)	25.9 (54.6)	28.7 (49.1)	34.6 (71.0)	44.0 (93.4)	33.0 (51.4)	42.1 (63.9)	− 3.8 (3.3)	0.25	3.8 (9.6)	0.698
Snacks, g, mean (SD)	23.1 (63.6)	19.3 (40.6)	13.0 (32.3)	9.3 (19.8)	24.8 (68.0)	23.7 (78.4)	12.4 (29.5)	9.8 (27.7)	2.7 (2.8)	0.35	5.7 (6.6)	0.384
Beverages, g, mean (SD)	8.7 (39.5)	7.3 (26.9)	4.9 (27.1)	2.5 (12.6)	7.3 (35.3)	10.7 (56.2)	5.1 (22.1)	6.4 (28.7)	− 1.4 (1.7)	0.402	0.4 (4.9)	0.936
**Physical activity/day**												
Vigorous activity, min, mean (SD)	32.0 (62.5)	45.0 (17.3)	24.2 (26.1)	22.1 (12.4)	29.9 (40.3)	21.6 (22.3)	20.5 (43.5)	16.0 (20.2)	1.5 (12.1)	0.900	− 15.6 (13.3)	0.253
Moderate activity, min, mean (S.D.)	21.0 (41.0)	12.4 (14.7)	15.9 (13.5)	19.6 (15.0)	24.8 (50.8)	38.3 (110.8)	15.8 (21.7)	16.5 (14.4)	4.0 (5.4)	0.46	33.1 (29.8)	0.271
Walking, min, mean (SD)	47.5 (41.7)	48.0 (36.7)	47.0 (38.7)	51.9 (35.3)	48.9 (44.6)	52.9 (46.1)	43.3 (37.4)	45.1 (68.4)	5.1 (2.3)	0.025	11.4 (7.0)	0.11

Abbreviations: *GDM* Gestational diabetes mellitus, *S.D.* standard deviation, *DID* the difference in difference, the follow-up is after 34 weeks of gestation, *Kcal*, *d* day, *g* Gram, *min* Minute

The intervention effect for all pregnant women or pregnant women with GDM was adjusted for hospital, and a statistical significance level of *P* < 0.05 (two-sided) was used for all pregnant women, while a significance level of *P* < 0.025 (with Bonferroni correction) was used for pregnant women with GDM

^**a**^DID value = follow-up (intervention − control) − baseline (intervention − control) and adjusted by hospital

### Antenatal care and blood glucose retests

Table 4 displays the antenatal care and blood glucose retest results for GDM women in both study groups. The actual number of blood glucose retests in GDM pregnant women was significantly higher in the intervention group than in the control group, with an IRR = 3.34 (95% CI, 1.61 to 6.93, *P* = 0.001) in the Poisson regression. In the intervention group, 68.9% of GDM women completed all the required glucose retests, whereas only 32.7% of GDM women in the control group completed all the required glucose retests, with an OR of 4.49 (95% CI: 1.34 to 15.0, *P* < 0.015). The blood glucose values at the first, second, and third retests for GDM women were lower in the intervention group than in the control group (Additional file 1: Fig. S3–S4).

**Table 4:** Blood glucose retests for participants with GDM between the intervention group and control group

	**Intervention**	**Control**	**Intervention effect for pregnant women with GDM** ^c^	
	GDM (*N* = 248)	GDM (*N* = 256)		
			**IRR (95% CI)**	***P*** ** value**
The number of blood glucose retests done for GDM pregnant women, mean (SD)^a^	1.5 (1.2)	0.4 (0.7)	3.34 (1.61 to 6.93)	0.001
			**OR (95% CI)**	***P*** ** value**
The number of GDM pregnant women completing all the blood glucose retests needed, No. (%)^b^	104 (41.9)	14 (5.5)	12.3 (3.14 to 48.4)	< 0.001
The number of GDM pregnant women completing at least one blood glucose retest, No. (%)^b^	171 (68.9)	83 (32.7)	4.49 (1.34 to 15.0)	< 0.015
The number of GDM pregnant women successfully controlling blood glucose by 34 weeks of gestation, *n*/No. (%)^b^	143/171 (83.6)	56/83 (67.4)	2.55 (1.04 to 6.24)	0.040
The number of GDM pregnant women successfully controlling blood glucose by delivery, *n*/No. (%)^b^	162/190 (85.3)	118/153 (77.1)	1.71 (0.83 to 3.52)	0.142

Abbreviations: *GDM* Gestational diabetes mellitus, *S.D.* Standard deviation, *OR* Odds ratio, *IRR* Incidence rate ratio

^a^Poisson regression model

^b^Generalized linear model with a logistic distribution link

^c^The intervention effect was adjusted for hospital, and a statistical significance level of *P* < 0.025 was used with Bonferroni correction for pregnant women with GDM

## Discussion

This multicenter RCT presented compelling evidence of the effectiveness of a targeted financial subsidy program for pregnant women and healthcare providers in reducing combined maternal and neonatal complications, particularly in a rural Chinese context. Notably, the subsidy program’s impact was primarily demonstrated through GDM management rather than screening, offering a new perspective on GDM control in China. The RCT design and randomization, combined with a large sample size of pregnant women, high retention rates, and specific settings from low-income rural areas of China, contributed to the study’s strengths. Overall, the findings of this RCT highlighted the effectiveness of financial subsidies in achieving better maternal and neonatal outcomes, with important implications for improving GDM management in resource-limited settings.

Screening for GDM was an essential initial step in managing the condition. A higher uptake rate indicated increased monitoring of potential GDM risks. Interestingly, we observed an increase in screening uptake in both the intervention and control groups, suggesting that the one-time out-of-pocket cost of screening might not be a significant barrier to GDM screening. Instead, regular screening reminders to participants in the control group played a vital role in pregnant women’s GDM screening uptake. This highlighted the importance of increasing awareness of GDM screening among pregnant women in low-income rural areas of China. This highlighted the importance of increasing awareness of GDM screening among pregnant women in low-income rural areas of China.

The RCT demonstrated that an economic subsidy program in low-income rural areas of China led to improved maternal and neonatal outcomes for pregnant women with GDM, primarily due to better glycemic control resulting from positive behavioral changes. Notably, there was an improvement in lifestyle habits, including dietary and physical activity patterns, which were the primary therapeutic strategies for managing GDM. While the individual components of lifestyle interventions remained unclear [32–34], Cochrane reviews demonstrated that they could reduce maternal and neonatal complications [32]. The primary aim of our study was to evaluate the impact of subsidies on maternal and neonatal complications associated with GDM, rather than to assess the individual effects of each aspect of GDM care. The effectiveness of various components of GDM screening and management care had fully been demonstrated in the previous literature. Another key aspect of the trial was providing the subsidy intervention to healthcare providers through financial compensation, motivating them to implement standardized GDM management. While quantifying the subsidy’s effects on healthcare providers’ behavior was challenging, its impact was reflected in improved dietary and physical activity patterns of pregnant women. Therefore, we recommended a subsidy for managing GDM, targeting both pregnant women and healthcare providers.

The RCT study demonstrated that the financial subsidy program not only improved maternal and neonatal outcomes but also positively impacted adherence to antenatal care and blood glucose retest rates among pregnant women in low-income rural areas of China. Regular antenatal care and blood glucose retests were crucial steps in GDM management, as they were associated with reduced maternal and neonatal complications. In the trial, healthcare providers regularly reminded pregnant women in the intervention group about the importance of antenatal care and glucose retests. Self-monitoring of glucose control during pregnancy was challenging, and highly intensive passive management by healthcare providers was recommended, as suggested by previous studies [1, 16, 35]. The study demonstrated that GDM women in the intervention group had higher glucose retest rates than those in the control group, leading to positive lifestyle changes and a deeper understanding of glucose control. Additionally, the results showed that the intervention group had better glucose control than the control group. Overall, the findings highlighted the importance of active and intensive management by healthcare providers in promoting adherence to antenatal care and blood glucose retests among pregnant women with GDM.

In addition to the reduction in combined complications, this subsidy study also observed a declining trend in the cesarean section (CS) rate. Over the past two decades, maternal and neonatal health in China significantly improved, primarily due to the widespread adoption of in-hospital delivery, as reported in previous studies [36, 37]. Beginning in 2009, subsidies were introduced to encourage in-hospital delivery for pregnant women in rural areas, resulting in 99.9% of newborns in low-income rural areas being delivered in hospitals by 2018 [37], but with a marked increase in CS rates [38]. However, this subsidy intervention trial for GDM care resulted in a 7% reduction in the CS rate for rural women with GDM, leading to improved maternal and neonatal health as well as reduced costs associated with CS. Although other maternal and neonatal outcomes such as premature delivery, gestational hypertension, and neonatal jaundice were also studied, their incidence rates were too low to detect differences due to limited sample sizes.

Finally, robust evidence from numerous RCTs affirming the efficacy of health education, lifestyle modifications, and pharmacotherapy in GDM care [22–27], their translation into practical, real-world applications often falls short of expectations [39]. Empirical studies consistently underscore the substantial economic burden borne by women managing GDM [39–41]. Remarkably, the literature lacks investigations into the potential role and impact of financial support mechanisms in the context of GDM management [41]. The present study, pioneering in this regard, aims to bridge this gap by providing initial insights and serving as a reference for future research. The findings emphasize the necessity for more inclusive and economically accessible GDM screening and management strategies, thereby paving the way for improved health outcomes and the broader applicability of these interventions.

The study exhibited several limitations. The first primarily stemmed from the use of individual-based randomization. This approach was necessary to address potential variations in maternal and neonatal complication rates, healthcare professionals’ awareness and practices, maternal lifestyle patterns, and hospital administrative protocols across the participating hospitals. While this study’s design effectively minimizes confounding factors, it forgoes the inherent advantages of cluster randomization, leading to the inability to maintain blinding between the intervention and control groups. Consequently, healthcare providers might have inadvertently intensified GDM screening and management efforts among the control group, potentially influencing their behavior. This could result in an improvement of GDM management in the control group, leading to a potential underestimation of the intervention’s true effectiveness. Despite this, the study still found that the intervention of GDM screening and management subsidy significantly reduced combined maternal and neonatal complications associated with GDM. Notably, the core intervention measure, the “subsidy,” was only provided to the intervention group. This ensured that the intervention group received free GDM screening and management care as part of the study intervention. The subsidy for the intervention group covered the costs of these services, and healthcare providers received compensation for providing GDM screening and management care. In contrast, the control group had to pay for these services out of pocket if they chose to receive them, without any subsidy. This differential provision of subsidies and services between the two groups reduced the likelihood of contamination and eliminated the possibility of switching between the groups. Second, the trial’s short duration meant that there was insufficient power to assess potential long-term benefits, such as the impact on the incidence of postpartum type II diabetes. Third, due to the study design, the subsidized program could not provide insulin therapy if necessary. Even if pregnant women might be transferred to upper-level hospitals for various reasons, including the need for medication management, their maternal and neonatal outcomes were still followed up. Fourth, the GDM screening uptake rates in the intervention and control groups were a little bit different (97.2% vs. 94.5%). We acknowledge that the different GDM screening uptake rates might have the potential to bias the results of the subgroup analysis focused specifically on the subgroup of participants with GDM. Due to limited space, we were unable to present the results of the cost-effectiveness analysis here, but they will be analyzed and presented later. The cost analyses included subsidies, medical supplies, labors, times, and others.

## Conclusions

This study provides robust evidence demonstrating the effectiveness of a subsidized GDM screening and management program in rural areas of China. The program effectively engaged both pregnant women and healthcare providers, promoting adherence to the intervention. This led to notable improvements in dietary patterns, increased physical activity, and enhanced blood glucose control in pregnant women, culminating in a significant reduction in maternal and neonatal complications. The implications of these findings are twofold: clinically, they underline the necessity for healthcare systems, especially in rural settings, to adopt similar GDM screening and management models. From a research perspective, the study lays the groundwork for future investigations into the long-term effects and cost-effectiveness of such interventions. Moreover, the results offer valuable insights for policy formulation, advocating for the integration of GDM care into public health strategies and emphasizing its potential to improving reproductive health outcomes in low-income areas. The study’s findings suggest that this program could be seamlessly incorporated into routine obstetric examinations, supporting the development of public health policies and programs that include GDM screening and management. This approach could foster a “pay-it-forward” system, enhancing reproductive health through accessible, affordable, and targeted financial interventions. Overall, the study not only enriches the existing literature on GDM screening and management but also provides practical guidance for clinicians, researchers, and policymakers dedicated to optimizing care in underserved populations.

### Supplementary Information

**Additional file1: Fig. S1.** Study sites. **Fig. S2.** Flowchart. **Fig. S3.** Fasting blood glucose values (mmol/L) in women diagnosed with GDM between the intervention group and control group (two-week interval). **Fig. S4.** The 2h-postprandial blood glucose (mmol/L) in women diagnosed with GDM between the intervention group and control group (two-week interval). **Table. S1.** Maternal and neonatal complications related to GDM. **Table. S2.** Baseline characteristics of pregnant women of included participants and those lost to follow-up. **Table. S3.** Effects of subsidy (intervention) on maternal and neonatal outcomes for overall women and GDM women. **Table. S4.** Effects of subsidy (intervention) on five common complications in pregnant women and newborns. **Table. S5.** Other complications (besides five common complications) in pregnant women and newborn. **Table. S6.** Maternal and neonatal complications between the intervention group and the control group adjusted by province. CONSORT guidelines CONSORT 2010 checklist of information to include when reporting a randomized trial. Supporting Material The protocol of lifestyle management for intervention group.

## Data Availability

The Data Management Group at the China Center for Health Development Research of Peking University (CCHDS-DMG) served as the Data and Safety Monitoring Board for this study, providing guidance throughout its implementation. The CCHDS-DMG has the authority to recommend the early termination of the trial if concerns regarding participant safety arise. Relevant anonymized patient-level data will be made available one year after the publication of the primary manuscript on request from the corresponding author. Request for data sharing will be handled in line with the relevant regulations for data access and sharing in China. It will need the approval of the trial steering committee, Peking University Health Science Center Review Board.

## Abbreviations

AEs: Adverse events
CIs: Confidence intervals
CS: Cesarean section
DID: Difference-in-difference
GDP: Gross domestic product
GDM: Gestational diabetes mellitus
IRR: Incidence rate ratio
OGTT: Oral glucose tolerance test
OR: Odds ratios
RCTs: Randomized controlled trials
SD: Standard deviation
