# Supplementary material for: Subsidized gestational diabetes mellitus screening and management program in rural China: a pragmatic multicenter, randomized controlled trial
Source: BMC Med. 2024 Mar 5;22:98. doi: 10.1186/s12916-024-03330-1 (PMC10916202; doi:10.1186/s12916-024-03330-1)
Supplement: Supplementary file 1 — Additional file1: Fig. S1. Study sites. Fig. S2. Flowchart. Fig. S3. Fasting blood glucose values (mmol/L) in women diagnosed with GDM between the intervention group and control group (two-week interval). Fig. S4. The 2h-postprandial blood glucose (mmol/L) in women diagnosed with GDM between the intervention group and control group (two-week interval). Table. S1. Maternal and neonatal complications related to GDM. Table. S2. Baseline characteristics of pregnant women of included participants and those lost to follow-up. Table. S3. Effects of subsidy (intervention) on maternal and neonatal outcomes for overall women and GDM women. Table. S4. Effects of subsidy (intervention) on five common complications in pregnant women and newborns. Table. S5. Other complications (besides five common complications) in pregnant women and newborn. Table. S6. Maternal and neonatal complications between the intervention group and the control group adjusted by province. CONSORT guidelines CONSORT 2010 checklist of information to include when reporting a randomized trial. Supporting Material The protocol of lifestyle management for intervention group. [file 12916_2024_3330_MOESM1_ESM.docx]

**Additional File 1**

**Fig. S1** Study sites

**Fig. S2** Flowchart

**Fig. S3** Fasting blood glucose values (mmol/L) in women diagnosed with GDM between the intervention group and control group (two-week interval)

**Fig. S4** The 2h-postprandial blood glucose (mmol/L) in women diagnosed with GDM between the intervention group and control group (two-week interval)

**Table. S1** Maternal and neonatal complications related to GDM

**Table. S2** Baseline characteristics of pregnant women of included participants and those lost to follow-up

**Table. S3** Effects of subsidy (intervention) on maternal and neonatal outcomes for overall women and GDM women

**Table. S4** Effects of subsidy (intervention) on five common complications in pregnant women and newborns

**Table. S5** Other complications (besides five common complications) in pregnant women and newborn

**Table. S6** Maternal and neonatal complications between the intervention group and the control group adjusted by province

**CONSORT guidelines** CONSORT 2010 checklist of information to include when reporting a randomized trial

**Supporting Material** The protocol of lifestyle management for intervention group

**Fig. S1** Study Sites

**
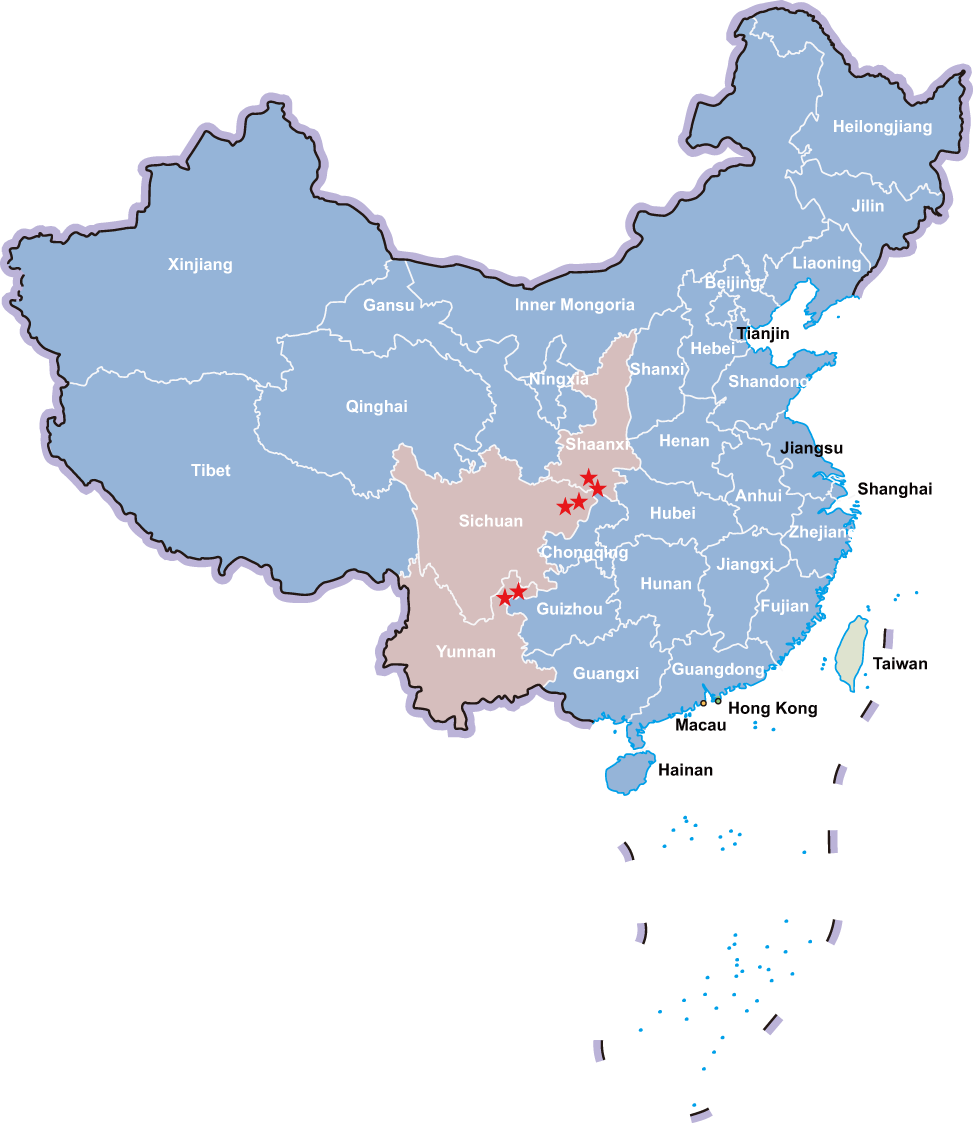
**

: Study sites

**Fig. S2** Flowchart

Participants were recruited in the 24-28th week of gestation. All participants signed informed written consent.

Demographic characteristics of eligible participants were obtained via a self-administered questionnaire, followed by random assignment to either the intervention or control group

Intervention group (subsided care)

Control group (usual care)

Before screening

1. Pregnant women underwent an individual session lasting ≥10 minutes, which covered various topics such as the concept of GDM, risk factors, complications, screening procedures, dietary and exercise interventions, recommended foods and recipes, physical activity, treatments, and precautions. The session was conducted in a private room, and doctors/nurses established a WeChat group for remote supervision and management.
2. A handbook containing the session's content was provided to each participant.
3. Pregnant women were offered free GDM screening and management services, including a OGTT voucher and 1-3 free glucose retest vouchers based on their gestational week.
4. Department was compensated 10 Chinese Yuan / per person for GDM education and glucose tests.

Screening

The medical team documented OGTT procedures, results, glucose retests, antenatal visits, and exams. Some pregnant women may have missed scheduled screenings. For non-compliance with OGTT, we recorded absence of screening.

Pregnant women were provided usual care without any subsidies but had the option to self-fund GDM screening and management. However, the WeChat group's remote supervision and management reminders were not accessible to them.

The medical team documented OGTT procedures, results, glucose retests, antenatal visits, and exams. Some pregnant women may have missed scheduled screenings. If the OGTT test was not taken, we recorded absence of screening.

Women with GDM received free services including：

1. Individual 30-minute sessions on diet and exercise every two weeks.
2. Weekly log books for recording data.
3. Remote consultation via WeChat for dietary and exercise advice.
4. Glucose retest vouchers, and 20 CNY compensation per medical staff member (doctor or nurse) for GDM lifestyle management during delivery.

After screening

Women with GDM

Normal women

Non-GDM diagnosed women received weekly dietary and exercise guidance (via WeChat) and more intensive instructions during monthly antenatal visits.

The healthcare personnel mandated to prompt and inform GDM patients for clinical appointments and prepare for glucose retesting through WeChat or phone communication.

Before retest

Doctors record the result of glucose retests, provide one-to-one instructions and adjust suggestions on individual diet and exercise according to women's logs.

Retest

Intervention group

Control group

At the 34th weeks of gestation, women completed self-administered questionnaires to assess GDM cognition, diet, and physical activity again. A final glucose test was conducted for all women prior to delivery. Maternal and neonatal outcomes were obtained from medical records for women who delivered in the trial hospital and via telephone for those who did not.

Women diagnosed with GDM were given standard care recommendations for diet and exercise. If they chose to pay for it themselves, they could also receive additional glucose retests, as well as continued support for diet and exercise. However, remote consultations via WeChat were not available as an option.

Women without a diagnosis of GDM were routinely monitored until delivery.

Normal women

Doctors provided regular consultations after self-paid glucose retests.

Delivery

Women with GDM

**Fig. S3** Fasting blood glucose values (mmol/L) in women diagnosed with GDM between the intervention group and control group (two-week interval)


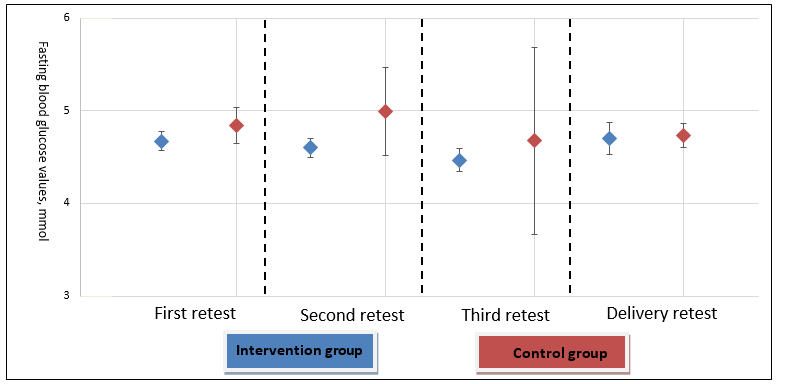


|  | First retest (mean,95%CI) | Second retest (mean,95%CI) | Third retest (mean,95%CI) | Delivery retest (mean,95%CI) |
| --- | --- | --- | --- | --- |
| Intervention group  (N=248) | 4.67 (4.56, 4.78)  n=163 | 4.60 (4.49, 4.70)  n=122 | 4.47 (4.34, 4.59)  n=77 | 4.70 (4.52, 4.87)  n=73 |
| Control group  (N=256) | 4.84 (4.64, 5.03)  n=81 | 4.99 (4.51, 5.46)  n=23 | 4.68 (3.67, 5.68)  n=7 | 4.73 (4.60, 4.86)  n=79 |

**Fig. S4** The 2h-postprandial blood glucose (mmol/L) in women diagnosed with GDM between the intervention group and control group (two-week interval)


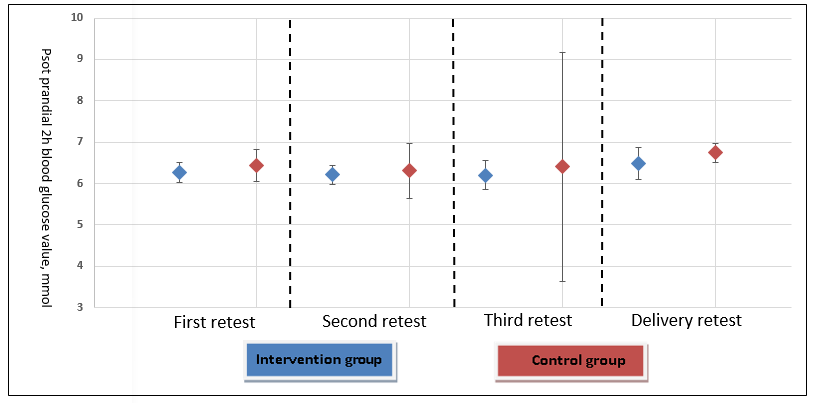


|  | First retest (mean,95%CI) | Second retest (mean,95%CI) |  | Third retest (mean,95%CI) | Delivery retest (mean,95%CI) |
| --- | --- | --- | --- | --- | --- |
| Intervention group  (N=248) | 6.25 (6.02, 6.49)  n=153 | 6.21 (5.98, 6.45)  n=115 |  | 6.20 (5.86, 6.55)  n=74 | 6.48 (6.09, 6.87)  n=57 |
| Control group  (N=256) | 6.43 (6.04, 6.82)  n=65 | 6.31 (5.64, 6.97)  n=14 |  | 6.41(3.64, 9.18)  n=5 | 6.74 (6.50, 6.98)  n=70 |

**Table. S1** Maternal and neonatal complications related to GDM

| **Item** | **Maternal complications** | **Fetal/neonatal complications** |
| --- | --- | --- |
| **1** | **Spontaneous abortions** | **Stillbirth:** Stillbirth is the delivery of a baby with no signs of life, known to have died after 24 weeks of pregnancy. |
| **2** | **Pre-Eclampsia** is defined as the new onset of hypertension and proteinuria after the 20th week of gestation. | **Neonatal death:** Neonatal deaths are defined as deaths among liveborn infants during the first 28 days of life |
| **3** | **Gestational hypertension:** Gestational hypertension is diagnosed when blood pressure readings are higher than 140/90 mm Hg in a woman who had normal blood pressure prior to 20 weeks and has no proteinuria (excess protein in the urine) | **Non-chromosomal congenital malformations** |
| **4** | **Amniotic fluid turbidity** | **Fetal macrosomia:** Birth weights ≥ 4000 g |
| **5** | **Polyhydramnios:** Polyhydramnios is defined as the deepest fluid pocket of more than 8 cm or an amniotic fluid index of at least 25 cm or more | **Low-birth-weight infants:** Birth weights＜2500 g |
| **6** | **Oligohydramnios:** Oligohydramnios is defined as <500 mL of amniotic fluid and is typically assessed by the amniotic fluid index (AFI, measurements of amniotic fluid pockets ≥2 cm) | **Neonatal asphyxia:** Newborns with progressive dyspnea and respiratory failure within 4-12 hours after birth, including progressive dyspnea, moaning, cyanosis, and inspiratory three concave signs. |
| **7** | **Intrauterine infection** | **Neonatal pneumonia** |
| **8** | **Fetal growth restriction:** Fetal size is abnormal and does not reach its genetic growth potential in utero | **Neonatal anaemia:** Venous hemoglobin (HB) w<130g / L (13g/dl) or capillary HB < 145g / L (14.5g / dl) 2 weeks after birth |
| **9** | **Placenta previa:** Placenta previa is defined as implantation within the lower uterine segment, with placental parenchyma completely covering the cervical os (complete Previa) or located near or partially covering the os (partial previa). | **Neonatal jaundice:** Neonatal jaundice in 28 days after birth |
| **10** | **Preterm labo**r is defined as regular uterine contractions occurring at least once every 10 minutes and resulting in cervical dilatation or effacement before 37 weeks gestation. | **Neonatal hypoglycemia:** Blood glucose < 1.7mmol/L in 3 days after birth, or < 2.2mmol/L after three days of birth for normal birth. blood glucose < 1.1mmol/L in 3 days after birth, or < 2.2mmol/L after 3days of birth for preterm birth. |
| **11** | **Postmature delivery:** More than 42 weeks of gestation | **Neonatal infection** |
| **12** | **Cesarean section** | **Fetal distress:** Foetal with hypoxia in the uterus. |
| **13** | **Premature rupture of membranes**: Premature rupture of membranes (PROM) is defined as the prelabor rupture of membranes before the expected date of childbirth. | **Neonatal thyroid abnormalities** |
| **14** | **Dystocia:** It is defined as difficult labor and delivery. | **Neonatal cholestasis** |
| **15** | **Postpartum hemorrhage:** Postpartum hemorrhage is defined as the loss of more than 500ml of blood after the completion of the delivery. |  |

**Table. S2** Baseline characteristics of pregnant women of included participants and missed follow-up to delivery participants.

|  | **Included (N=3294)** | | | | **Missed (N=106)** | | | | |
| --- | --- | --- | --- | --- | --- | --- | --- | --- | --- |
|  | **Intervention** | | **Control** | | **Intervention** | | **Control** | | |
|  | **Overall**  **(N=1649)** | **GDM**  **(N=248)** | **Overall**  **(N=1645)** | **GDM**  **(N=256)** | **Overall**  **(N=56)** | **GDM**  **(N=8)** | **Overall**  **(N=50)** | **GDM**  **(N=7)** |  |
| Fasting blood glucose, mmol/L, mean (SD) | 4.4 (0.6) | 5.2 (0.7) | 4.5 (0.6) | 5.3 (0.6) | 4.6 (0.6) | 5.5 (0.6) | 4.8 (0.5) | 5.5 (0.4) |  |
| One hour blood glucose, mmol/L, mean (SD) | 6.8 (1.8) | 8.9 (2.2) | 7.0 (1.8) | 9.2 (2.2) | 6.9 (1.6) | 7.5 (2.0) | 8.6 (1.9) | 10.3 (2.5) |  |
| Two-hour blood glucose, mmol/L, mean (SD) | 6.0 (1.4) | 7.6 (2.0) | 6.2 (1.4) | 7.7 (1.7) | 5.9 (1.09) | 6.8 (1.6) | 7.1 (1.5) | 8.0 (2.2) |  |
| Maternal age, *years, mean (SD)* | 27.0 (5.0) | 28.6 (5.4) | 27.2 (5.0) | 29.1 (5.5) | 26.1 (5.6) | 27.1 (8.9) | 25.1 (4.3) | 23.3 (5.9) |  |
| Gestational weeks at recruitment, weeks*, mean (SD)* | 25.4 (1.5) | 25.6 (1.5) | 25.4 (1.5) | 25.7 (1.7) | 25.8 (1.9) | 25.6 (1.1) | 25.7 (2.3) | 26.9 (2.0) |  |
| Pre-pregnancy BMI, *kg/m^2^, mean (SD)* | 21.1 (3.7) | 22.5 (4.3) | 21.2 (3.3) | 22.1 (3.8) | 23.6 (7.9) | 24.1 (3.2) | 23.5 (7.2) | 20.2 (3.7) |  |
| Systolic pressure before pregnancy, *mmHg, mean (SD)* | 110.0 (11.5) | 113 (12.0) | 110.2 (11.4) | 112.0 (12.9) | 117.1 (13.0) | 124.7 (12.9) | 110.8 (8.6) | 118.0 (8.5) |  |
| Diastolic pressure before pregnancy, *mmHg, mean (SD)* | 68.5 (10.9) | 69.6 (8.6) | 68.1 (10.2) | 69.5 (10.0) | 66.5 (9.6) | 68.0 (10.1) | 67.0 (6.6) | 77.0 (9.8) |  |
| Systolic pressure before delivery, *mmHg, mean (SD)* | 112.5 (11.5) | 113 (12.7) | 112.1 (11.7) | 112.8 (12.4) | 119.8 (13.1) | 113.3 (11.5) | 103.3 (5.8) | .. |  |
| Diastolic pressure before delivery, *mmHg, mean (SD)* | 70.3 (9.3) | 70.9 (9.3) | 70.0 (9.4) | 71.1 (9.7) | 76.1 (10.2) | 76.3 (5.5) | 66.0 (5.3) | .. |  |
| Gestational weeks at birth, weeks, mean (SD) | 39.1 (1.5) | 39.1 (1.6) | 39.1 (1.6) | 39.0 (1.7) | .. | .. | .. | .. |  |
| Birthweight, kg, mean (SD) | 3.2 (0.4) | 3.3 (0.4) | 3.2 (0.4) | 3.3 (0.4) | .. | .. | .. | .. |  |
| Primiparous, *No. (%)* | 500 (33.8) | 62 (28.3) | 491 (34.7) | 63 (29.03) | 16 (37.2) | 3 (42.9) | 12 (40.0) | 2 (50.0) |  |
| Education, *No. (%)* |  |  |  |  |  |  |  |  |  |
| below primary school | 23 (1.5) | 7 (3.1) | 18 (1.2) | 4 (1.8) | .. | .. | 1 (3.2) | .. |  |
| Primary school | 180 (11.9) | 29 (12.7) | 155 (10.7) | 31 (14.0) | 12 (30.2) | .. | 2 (6.5) | .. |  |
| Junior high school | 738 (48.6) | 101 (44.3) | 670 (46.1) | 93 (41.9) | 16 (37.2) | 3 (50.0) | 13 (41.9) | 2 (50.0) |  |
| Senior high school | 166 (10.9) | 22 (9.6) | 166 (11.4) | 27 (12.1) | 3 (7.0) | 1 (16.7) | 7 (22.6) | 1 (25.0) |  |
| Technical secondary school | 132 (8.7) | 17 (7.5) | 128 (8.8) | 16 (7.2) | 3 (7.0) | 2 (33.3) | 3 (9.7) | 1 (25.0) |  |
| Two year’s college | 168 (11.0) | 26 (11.4) | 177 (12.2) | 28 (12.6) | 4 (9.3) | .. | 1 (3.2) | .. |  |
| Four years of university or above | 112 (7.4) | 26 (11.4) | 139 (9.6) | 23 (10.4) | 4 (9.3) | .. | 4 (12.9) | .. |  |
| Family income, ten thousand CNY/ year, No. (%) |  |  |  |  |  |  |  |  |  |
| Mean (SD). | 5.9 (4.8) | 5.7 (3.5) | 6.2 (6.8) | 6.5 (5.3) | 5.1 (4.4) | 8.5 (5.8) | 6.9 (4.4) | 10 (7.1) |  |
| Median (*the interquartile range*) | 5.0 (3;8) | 5.0 (3;8) | 5.0 (3;8) | 5.0 (3;8) | 3.5 (1.3;9) | 9 (5;13) | 5 (4;9) | 10(5;15) |  |

Missed follow-up: Women without maternal and neonatal delivery outcomes; GDM: Gestational Diabetes Mellitus. SD: Standard Deviation; CNY: Chinese Yuan.

**Table. S3** Effects of subsidy (intervention) on maternal and neonatal outcomes for overall women and GDM women

|  | **Intervention Group** | | | **Control Group** | | | | **Intervention effect for all pregnant women*** | | | **Intervention effect for pregnant women with GDM*** | |
| --- | --- | --- | --- | --- | --- | --- | --- | --- | --- | --- | --- | --- |
|  | Overall (N=1,649) | Normal (n=1,354) | GDM (n=248) | | Overall (N=1,645) | Normal (n=1,299) | GDM (n=256) | | OR (95% CI) | *P* value | OR (95% CI) | *P* value |
| **Maternal complications**, No. (%) | 538 (33.8) | 442 (33.7) | 84 (35.0) | | 567 (35.6) | 441 (34.5) | 103 (41.4) | | 0.92 (0.84 to 1.01) | 0.066 | 0.76 (0.49 to 1.18) | 0.224 |
| **Neonatal complications**, No. (%) | 181 (11.4) | 150 (11.4) | 26 (10.8) | | 215 (13.5) | 170 (13.3) | 35 (14.1) | | **0.82 (0.76 to 0.88)** | **0.000** | 0.74 (0.45 to 1.20) | 0.227 |
|  |  |  |  | |  |  |  | | IRR (95% CI) |  | IRR (95% CI) |  |
| **The number of maternal complications per person, mean (SD)** | 0.37 (0.57) | 0.37 (0.57) | 0.40(0.60) | | 0.39(0.55) | 0.38 (0.55) | 0.50(0.56) | | 0.97 (0.89 to 1.05) | 0.442 | 0.88 (0.72 to 1.09) | 0.272 |
| **The number of neonatal complications per person, mean (SD)** | 0.12 (0.36) | 0.12 (0.36) | 0.11(0.34) | | 0.15(0.40) | 0.15 (0.40) | 0.15(0.37) | | **0.83 (0.79 to 0.87)** | **0.000** | 0.78 (0.48 to 1.27) | 0.331 |

*Abbreviation*: GDM, gestational diabetes mellitus; OR, odds ratio; IRR, incidence rate ratio.

We employed generalized linear regression models with a logistic distribution link for analyzing maternal complication and neonatal complications, and a Poisson regression model was utilized for assessing the number of maternal complications per person and the number of neonatal complications per person .*The intervention effect was adjusted by hospital, and statistical significance was set at P<0.05 with two-sided for all pregnant women and P<0.025 with Bonferroni correction for pregnant women with GDM.

**Table. S4** Effects of subsidy (intervention) on five common complications in pregnant women and newborns

|  | | | | | | | | | | |
| --- | --- | --- | --- | --- | --- | --- | --- | --- | --- | --- |
|  | **Intervention Group** | | | **Control Group** | | | **Intervention effect for all pregnant women*** | | **Intervention effect for pregnant women with GDM*** | |
|  | Overall (N=1,649) | Normal (n=1,354) | GDM (n=248) | Overall (N=1,645) | Normal (n=1,299) | GDM (n=256) | OR (95% CI) | *P* value | OR (95% CI) | *P* value |
| **Maternal complications**, No. (%) |  |  |  |  |  |  |  |  |  |  |
| Cesarean section | 474 (29.7) | 393 (29.9) | 72 (30.0) | 505 (31.7) | 396 (30.9) | 92 (37.0) | 0.91 (0.82 to 1.01) | 0.085 | 0.73 (0.50 to 1.05) | 0.094 |
| Premature delivery | 48 (3.0) | 37 (2.8) | 10 (4.2) | 63 (4.0) | 43 (3.4) | 13 (5.2) | 0.76 (0.66 to 0.86) | 0.000 | 0.78 (0.49 to 1.25) | 0.320 |
| Gestational hypertension | 22 (1.4) | 16 (1.2) | 6 (2.5) | 16 (1.0) | 13 (1.0) | 3 (1.2) | 1.38 (0.75 to 2.52) | 0.294 | 2.10 (0.78 to 5.63) | 0.140 |
| Postpartum hemorrhage | 15 (0.9) | 8 (0.6) | 6 (2.1) | 16 (1) | 13 (1.0) | 2 (0.8) | 0.94 (0.30 to 2.92) | 0.912 | 3.16 (1.39 to 7.19) | 0.006 |
| Premature rupture of membranes | 14 (0.9) | 12 (0.9) | 0 (0.0) | 7 (0.4) | 7 (0.5) | 0(0.0) | 2.01 (1.37 to 2.92) | 0.000 | .. | .. |
| **Neonatal complications**, No. (%) |  |  |  |  |  |  |  |  |  |  |
| Fetal macrosomia | 52 (3.3) | 43 (3.3) | 9 (3.8) | 65 (4.1) | 49 (3.8) | 14 (5.6) | 0.79 (0.56 to 1.12) | 0.193 | 0.65 (0.15 to 2.80) | 0.568 |
| Neonatal jaundice | 52 (3.3) | 44 (3.4) | 7 (2.9) | 64 (4.0) | 52 (4.2) | 10 (4.0) | 0.81 (0.76 to 0.85) | 0.000 | 0.72 (0.38 to 1.33) | 0.296 |
| Pneumonia of newborn | 16 (1.0) | 11 (0.9) | 4 (1.8) | 22 (1.4) | 15 (1.2) | 5 (2.0) | 0.72 (0.69 to 0.75) | 0.000 | 0.82 (0.72 to 0.94) | 0.007 |
| Low birth weight | 46 (2.9) | 39 (3.0) | 5 (2.1) | 49 (3.07) | 40 (3.1) | 4 (1.6) | 0.94 (0.73 to 1.20) | 0.613 | 1.30 (0.26 to 6.40) | 0.744 |
| Fetal distress in the uterus | 11 (0.7) | 9 (0.7) | 1 (0.4) | 15 (0.9) | 12 (0.9) | 3 (1.2) | 0.73 (0.58 to 0.92) | 0.007 | 0.34 (0.13 to 0.91) | 0.031 |

*Abbreviation*: GDM, gestational diabetes mellitus; OR, odds ratio.

We employed generalized linear regression models with a logistic distribution link for analyzing five common complications in pregnant women and newborns. *The intervention effect was adjusted by hospital, and statistical significance was set at P<0.05 with two-sided for all pregnant women and P<0.025 with Bonferroni correction for pregnant women with GDM.

**Table. S5** Other complications (besides five common complications) in pregnant women and newborns

|  | **Intervention Group (free GDM screening and Lifestyle Management)** | | | **Control Group (pregnant women need to pay for GDM screening and lifestyle management)** | | |  |
| --- | --- | --- | --- | --- | --- | --- | --- |
|  | **Overall (N=1649)** | **Normal**  **(N=1354)** | **GDM (N=248)** | **Overall (N=1645)** | **Normal**  **(N=1299)** | **GDM (N=256)** |  |
| **Maternal complications** |  |  |  |  |  |  |  |
| Placenta previa, No. (%) | 3 (0.2) | 3 (0.2) | 0 (0.0) | 0 (0.0) | 0 (0.0) | 0 (0.0) |  |
| spontaneous abortion, No. (%) | 2 (0.1) | 2 (0.2) | 0 (0.0) | 2 (0.1) | 1 (0.1) | 1 (0.4) |  |
| Amniotic fluid turbidity, No. (%) | 4 (0.2) | 4 (0.3) | 0 (0.0) | 0 (0.0) | 0 (0.0) | 0 (0.0) |  |
| Polyhydramnios, No. (%) | 0 (0.0) | 0 (0.0) | 0 (0.0) | 1 (0.1) | 1 (0.1) | 0 (0.0) |  |
| Oligohydramnios, No. (%) | 8 (0.5) | 8 (0.6) | 0 (0.0) | 4 (0.3) | 4 (0.3) | 0 (0.0) |  |
| Postmature delivery, No. (%) | 10 (0.6) | 10 (0.7) | 0 (0.0) | 7 (0.4) | 6 (0.5) | 1 (0.4) |  |
| Intrauterine infection, No. (%) | 1 (0.1) | 1 (0.1) | 0 (0.0) | 0 (0.0) | 0 (0.0) | 0 (0.0) |  |
| Fetal growth restriction, No. (%) | 1 (0.1) | 1 (0.1) | 0 (0.0) | 0 (0.0) | 0 (0.0) | 0 (0.0) |  |
| Severe eclampsia, No. (%) | 3 (0.2) | 2 (0.2) | 1 (0.4) | 0 (0.0) | 0 (0.0) | 0 (0.0) |  |
| Dystocia, No. (%) | 2 (0.1) | 1 (0.1) | 1 (0.4) | 5 (0.3) | 5 (0.4) | 0 (0.0) |  |
| **Neonatal complications** |  |  |  |  |  |  | |
| Neonatal hypoglycemia, No. (%) | 1 (0.1) | 1 (0.1) | 0 (0.0) | 0 (0.0) | 0 (0.0) | 0 (0.0) | |
| Neonatal infection, No. (%) | 1 (0.1) | 1 (0.1) | 0 (0.0) | 2 (0.1) | 2 (0.1) | 0 (0.0) | |
| Neonatal death, No. (%) | 3 (0.2) | 3 (0.2) | 0 (0.0) | 0 (0.0) | 0 (0.0) | 0 (0.0) | |
| Neonatal meningitis, No. (%) | 0 (0.0) | 0 (0.0) | 0 (0.0) | 1 (0.1) | 1 (0.1) | 0 (0.0) | |
| Neonatal Cholestasis, No. (%) | 3 (0.2) | 3 (0.2) | 0 (0.0) | 1 (0.1) | 0 (0.0) | 1 (0.4) | |
| Neonatal anemia, No. (%) | 3 (0.2) | 2 (0.2) | 1 (0.4) | 2 (0.2) | 2 (0.2) | 0 (0.0) | |
| Stillbirth, No. (%) | 2 (0.1) | 2 (0.1) | 0 (0.0) | 7 (0.4) | 7 (0.5) | 0 (0.0) | |
| Non-chromosomal congenital malformations, No. (%) | 4 (0.2) | 4 (0.3) | 0 (0.0) | 0 (0.0) | 0 (0.0) | 0 (0.0) | |

GDM: Gestational Diabetes Mellitus. SD: Standard Deviation. CI: Confidence Interval

There is not enough power to detect the differences in these complications between the intervention group and control group.

**Table. S6** Maternal and neonatal complications between the intervention group and the control group adjusted by province.

|  | **Intervention effect for all pregnant women*** | | | | |
| --- | --- | --- | --- | --- | --- |
|  | **Yunnan province** | | **Sichuan province** | **Shanxi’ province** | |
|  | OR (95% CI) | *P* value | OR (95% CI). *P* value | OR (95% CI) | *P* value |
| Maternal or neonatal complications | 0.84 (0.83 to 0.85) | 0.000 | 1.14 (0.75 to 1.75) 0.537 | 0.83 (0.83 to 0.84) | 0.000 |
|  | IRR (95%CI) |  | IRR (95%CI) | IRR (95% CI) |  |
| The number of maternal and neonatal complications per person | 0.90 (0.87 to 0.92) | 0.000 | 1.09 (0.94 to 1.25) 0.219 | 0.91 (0.88 to 94) | 0.000 |

*Abbreviation*: GDM, gestational diabetes mellitus; OR, odds ratio; IRR, incidence rate ratio.

We employed generalized linear regression models with a logistic distribution link for analyzing maternal and neonatal complications, and a Poisson regression model was utilized for assessing the number of maternal and neonatal complications per person.

**CONSORT guideline**

**
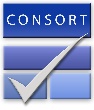
CONSORT 2010 checklist of information to include when reporting a randomized trial***

| **Section/Topic** | **Item No** | **Checklist item** | **Reported on page No** |
| --- | --- | --- | --- |
| **Title and abstract** | | | |
|  | 1a | Identification as a randomised trial in the title | 1 |
|  | 1b | Structured summary of trial design, methods, results, and conclusions (for specific guidance see CONSORT for abstracts) | 2-4 |
| **Introduction** | | | |
| Background and objectives | 2a | Scientific background and explanation of rationale | 5-7 |
|  | 2b | Specific objectives or hypotheses | 7,8 |
| **Methods** | | | |
| Trial design | 3a | Description of trial design (such as parallel, factorial) including allocation ratio | 8-10 |
|  | 3b | Important changes to methods after trial commencement (such as eligibility criteria), with reasons | No substantial changes |
| Participants | 4a | Eligibility criteria for participants | 9,10 |
|  | 4b | Settings and locations where the data were collected | 8 |
| Interventions | 5 | The interventions for each group with sufficient details to allow replication, including how and when they were actually administered | 10-14 |
| Outcomes | 6a | Completely defined pre-specified primary and secondary outcome measures, including how and when they were assessed | 14,15 |
|  | 6b | Any changes to trial outcomes after the trial commenced, with reasons | No substantial changes |
| Sample size | 7a | How sample size was determined | 16,15 |
|  | 7b | When applicable, explanation of any interim analyses and stopping guidelines | No interim analysis |
| Randomisation: |  |  |  |
| Sequence generation | 8a | Method used to generate the random allocation sequence | 10 |
|  | 8b | Type of randomisation; details of any restriction (such as blocking and block size) | 10 |
| Allocation concealment mechanism | 9 | Mechanism used to implement the random allocation sequence (such as sequentially numbered containers), describing any steps taken to conceal the sequence until interventions were assigned | 10 |
| Implementation | 10 | Who generated the random allocation sequence, who enrolled participants, and who assigned participants to interventions | 10 |
| Blinding | 11a | If done, who was blinded after assignment to interventions (for example, participants, care providers, those assessing outcomes) and how | 10 |
|  | 11b | If relevant, description of the similarity of interventions | 11 |
| Statistical methods | 12a | Statistical methods used to compare groups for primary and secondary outcomes | 16-18 |
|  | 12b | Methods for additional analyses, such as subgroup analyses and adjusted analyses | 17,18 |
| **Results** | | | |
| Participant flow (a diagram is strongly recommended) | 13a | For each group, the numbers of participants who were randomly assigned, received intended treatment, and were analysed for the primary outcome | 18 |
|  | 13b | For each group, losses and exclusions after randomisation, together with reasons | 18 |
| Recruitment | 14a | Dates defining the periods of recruitment and follow-up | 18 |
|  | 14b | Why the trial ended or was stopped | Figure 1 |
| Baseline data | 15 | A table showing baseline demographic and clinical characteristics for each group | Table 1 |
| Numbers analysed | 16 | For each group, number of participants (denominator) included in each analysis and whether the analysis was by original assigned groups | Figure 1 |
| Outcomes and estimation | 17a | For each primary and secondary outcome, results for each group, and the estimated effect size and its precision (such as 95% confidence interval) | Table 2-4 |
|  | 17b | For binary outcomes, presentation of both absolute and relative effect sizes is recommended | Table 2 |
| Ancillary analyses | 18 | Results of any other analyses performed, including subgroup analyses and adjusted analyses, distinguishing pre-specified from exploratory | Table 2-4; supplementary Table 3-6 |
| Harms | 19 | All important harms or unintended effects in each group (for specific guidance see CONSORT for harms) | 19,20 |
| **Discussion** | | | |
| Limitations | 20 | Trial limitations, addressing sources of potential bias, imprecision, and, if relevant, multiplicity of analyses | 25,26 |
| Generalisability | 21 | Generalisability (external validity, applicability) of the trial findings | 26 |
| Interpretation | 22 | Interpretation consistent with results, balancing benefits and harms, and considering other relevant evidence | 21-26 |
| **Other information** | | |  |
| Registration | 23 | Registration number and name of trial registry | 4 |
| Protocol | 24 | Where the full trial protocol can be accessed, if available | 8 |
| Funding | 25 | Sources of funding and other support (such as supply of drugs), role of funders | 4 |

*We strongly recommend reading this statement in conjunction with the CONSORT 2010 Explanation and Elaboration for important clarifications on all the items. If relevant, we also recommend reading CONSORT extensions for cluster randomised trials, non-inferiority and equivalence trials, non-pharmacological treatments, herbal interventions, and pragmatic trials. Additional extensions are forthcoming: for those and for up to date references relevant to this checklist, see [www.consort-statement.org](http://www.consort-statement.org).

**Supporting Material** The protocol of lifestyle management for intervention group

Participants in the intervention group will receive free GDM screening, and an OGTT (75g) will be administered to all women between 24 and 28 weeks of gestation using the diagnostic criteria recommended by the IADPSG, namely, fasting venous glucose of 5.1mmol/L or higher, 1-hour venous glucose of 10.0mmol/L or higher, 2-our venous glucose of 8.5mmol/L or higher or a combination of these.33 For women diagnosed with GDM in the intervention group, a free of charge 30min individual session on diet and exercise management and antenatal retest.of glucose will subsequently be provided every 2weeks until their glucose becomes normal. A nurse or doctor will be required to provide remote consultation and management of diet and exercise to women with GDM to promote the effect of self-monitoring every week by WeChat, and a log book will be used to record their daily

diets and exercise (online supplemental file 3), which is useful to help doctors tailor advice for each woman with GDM at retest. If a participant misses her retest, a nurse or doctor will contact and inform her again via telephone or WeChat. Up to three free retests for GDM will be provided according to women’s gestational weeks. The medical department was subsidised with ¥20 per participant for the provision of the GDM lifestyle management. For women without GDM, the remote consultation and management of diet and exercise will be provided every week by WeChat in case of abnormal glucose in the last trimester. Individual recommendations for diet and exercise will be based on the guidelines of diet and exercise for women with GDM,34 and will also be adjusted according to participants’ habitual preferences. Dietary recommendations will be based on the following principles: restricting dietary intake of saturated fat and exchanging carbohydrate-rich foods with a medium-to-high glycaemic index for foods with a lower glycaemic index to reduce the glycaemic load. For advice on physical activity,34 we will focus on incremental increases in walking tailored to the woman’s pre-existing activities. Moderate-intensity activities will be suggested, and additional options, including aerobics, yoga and swimming, will be encouraged, particularly for women already engaging in some physical activities. A medical record (online supplemental file 4) will also be used to record the effect of subsidised GDM screening and lifestyle management at delivery. After 34 weeks of gestation, all women in the intervention group will be surveyed again. Data on knowledge of GDM, diet, physical activities and quality of life will be collected via questionnaire 2 (online supplemental file 5).
